# The effect of thioredoxin and prochymosin coexpression on the refolding of recombinant alpaca chymosin

**DOI:** 10.18699/VJGB-23-50

**Published:** 2023-07

**Authors:** S.V. Belenkaya, D.N. Shcherbakov, A.I. Chapoval, T.I. Esina, V.V. Elchaninov

**Affiliations:** State Research Center of Virology and Biotechnology “Vector”, Rospotrebnadzor, Koltsovo, Novosibirsk region, Russia Altai State University, Barnaul, Russia; State Research Center of Virology and Biotechnology “Vector”, Rospotrebnadzor, Koltsovo, Novosibirsk region, Russia Altai State University, Barnaul, Russia; Altai State University, Barnaul, Russia; State Research Center of Virology and Biotechnology “Vector”, Rospotrebnadzor, Koltsovo, Novosibirsk region, Russia; Federal Altai Scientific Center for Agrobiotechnology, Siberian Research Institute of Cheesemaking, Barnaul, Russia

**Keywords:** thioredoxin (Trx), recombinant chymosin (rChn), inclusion bodies, milk-clotting activity, renaturation, тиоредоксин (Trx), рекомбинантный химозин (rChn), тельца включения, молокосвертывающая активность, ренатурация

## Abstract

The milk-clotting enzyme chymosin is a member of the group of aspartate proteinases. Chymosin is the main component of rennet traditionally obtained from the stomachs of dairy calves and widely used to coagulate milk in the production of various types of cheese. Another source of chymosin, which does not require the killing of animals, is based on recombinant DNA technology. Recombinant alpaca chymosin has a number of valuable technological properties that make it attractive for use in cheese-making as an alternative to recombinant bovine chymosin. The purpose of this work is to study the effect of coexpression of thioredoxin and prochymosin on the refolding of the recombinant zymogen and the activity of alpaca chymosin. To achieve this goal, on the basis of the pET32a plasmid, an expression vector was constructed containing the thioredoxin A gene fused to the N-terminal sequence of the marker enzyme zymogen, alpaca prochymosin. Using the constructed vector, pET-TrxProChn, a strain-producer of the recombinant chimeric protein thioredoxin-prochymosin was obtained. The choice of prochymosin as a model protein is due to the ability of autocatalytic activation of this zymogen, in which the pro-fragment is removed, together with the thioredoxin sequence attached to it, with the formation of active chymosin. It is shown that Escherichia coli strain BL21 transformed with the pET-TrxProChn plasmid provides an efficient synthesis of the thioredoxin-prochymosin chimeric molecule. However, the chimeric protein accumulates in inclusion bodies in an insoluble form. Therefore, a renaturation procedure was used to obtain the active target enzyme. Fusion of thioredoxin capable of disulfide-reductase activity to the N-terminal sequence of prochymosin provides optimal conditions for zymogen refolding and increases the yield of recombinant alpaca chymosin immediately after activation and during long-term storage by 13 and 15 %, respectively. The inclusion of thioredoxin in the composition of the chimeric protein, apparently, contributes to the process of correct reduction of disulfide bonds in the prochymosin molecule, which is reflected in the dynamics of the increase in the milk-clotting activity of alpaca chymosin during long-term storage.

## Abstract (ru)

Молокосвертывающий фермент химозин является представителем группы аспартатных протеиназ. Химозин – основной компонент сычужного фермента, традиционно получаемого из желудков телят-молокопоек и широко используемого для свертывания молока при производстве разнообразных видов сыра. Другой источник химозина, не требующий умерщвления животных, базируется на технологии рекомбинантных ДНК. Рекомбинантный химозин альпака обладает рядом ценных технологических свойств, делающих его привлекательным для использования в сыроделии в качестве альтернативы рекомбинантному химозину коровы. Цель настоящей работы – исследование влияния коэкспрессии тиоредоксина и прохимозина на рефолдинг рекомбинантного зимогена и активность химозина альпака. Для достижения поставленной цели на основе плазмиды pET32a был сконструирован экспрессионный вектор, содержащий ген тиоредоксина А, слитый с N-концевой последовательностью зимогена маркерного фермента – прохимозина альпака. С помощью сконструированного вектора pET-TrxProChn создан штамм-продуцент рекомбинантного химерного белка тиоредоксин-прохимозин. Выбор прохимозина в качестве модельного белка обусловлен способностью этого зимогена к автокаталитической активации, при которой происходит удаление профрагмента вместе с присоединенной к нему последовательностью тиоредоксина с образованием активного химозина. Показано, что штамм Escherichia coli BL21, трансформированный плазмидой pET-TrxProChn, обеспечивает эффективный синтез химерной молекулы тиоредоксин-прохимозин. Однако химерный белок накапливается в тельцах включения в нерастворимой форме. Поэтому для создания активного целевого фермента была использована процедура ренатурации. Присоединение тиоредоксина, обладающего способностью восстанавливать дисульфидные связи, к N-концевой последовательности прохимозина обеспечивает оптимальные условия для рефолдинга зимогена и увеличивает выход рекомбинантного химозина альпака сразу после активации и при длительном хранении на 13 и 15 % соответственно. Включение тиоредоксина в состав химерного белка, по всей видимости, способствует процессу корректного восстановления дисульфидных связей в молекуле прохимозина, что отражается на динамике роста молокосвертывающей активности химозина альпака при длительном хранении.

## Introduction

The development of genetic engineering and biotechnology methods makes it possible to obtain a plethora of technological and therapeutic proteins not from raw natural materials, but by synthesizing their recombinant analogs in various expression systems. Prokaryotes use for producing recombinant proteins is well studied and has important features such as rapid growth, a high level of protein synthesis in addition to a simple culturing protocol. Even though prokaryotes do not perform some post-translational modifications (glycosylation, phosphorylation, proteolytic processing) of synthesized protein as eukaryotes, these expression systems are used for industrial-scale production of various recombinant proteins (Huang et al., 2012; Rosano, Ceccarelli, 2014; Baeshen et al., 2015). This method for recombinant protein production provides a more simple and cost-effective approach compared to obtaining and maintaining stable eukaryotic producers (Rosano et al., 2019). The Escherichia coli expression system was used in our laboratory to produce and characterize new milk-clotting enzymes encoded by alpaca and maral chymosins genes (Belenkaya et al., 2018, 2020a, b).

When a heterologous protein is expressed in the E. coli system, there are several scenarios for its final localization: the synthesis and transport of the native product into periplasmic space; the accumulation in an inactive (aggregated) state in inclusion bodies; accumulation of the target recombinant protein in the cytoplasm and sometimes secretion into the culture medium (Baneyx, 1999). In the systems using strong promoters (such as the T7 promoter) and the corresponding E. coli strains, proteins usually accumulate in inclusion bodies and can be extracted with denaturing and chaotropic agents. To restore the native conformation of the denatured protein obtained from inclusion bodies, the renaturation (refolding) procedure is required. The key point of refolding is the gradual removal of denaturing and chaotropic components from the solution.

In systems with reversed micelles described earlier (Sakono et al., 2004), refolding can be carried out using dialysis, gel filtration (Li et al., 2004) or adsorption chromatography (Nara et al., 2009). Removal of the denaturating or chaotrope agents does not always lead to the restoration of the target product’s native structure. To increase the efficiency of renaturation, attempts were made to use proteins with chaperone activity (Wei et al., 2000; Rosano, Ceccarelli, 2014). Construction of a recombinant protein containing a whole or a part of chaperon protein followed by the chaperone separation from the target product can be an effective strategy (Li, Sousa, 2012; Emamipour et al., 2019; Rosano et al., 2019).

One of the widely used proteins with chaperone activity is thioredoxin (Trx). Thioredoxin is a product of trxA gene, a small enzyme that exhibits chaperone properties. It is important to note that this protein also has disulfide-reductase activity that participates in reversible oxidation of cysteine SH-groups to disulfide. In the case of Chn – chymosin (s), this is of particular importance because it contains three disulfide bridges and their correct closure is important for its enzymatic activity (Chen et al., 2000). Coexpression of Trx and the target protein enhances production, accelerates correct folding, increases solubility, and improves the functional properties of some cysteine-containing proteins and polypeptides such as: scFv antibodies (Jurado et al., 2006), Balanus albicostatus adhesive protein (Balcp19k) (Liang et al., 2015), full-length and fragmented tissue plasminogen activator (Bessette et al., 1999). We hypothesized that the expression of a chimeric molecule containing the thioredoxin and prochymosin sequence (Trx-ProChn) may affect its localization and refolding.

Chymosin (EC 3.4.23.4), an aspartic endopeptidase, is widely used in the cheese-making industry for curdling milk and producing a milk curd. The native structure of chymosin (Chn) is stabilized by three -S-S- bridges, which is an important process during renaturation of recombinant protein produced in the E. coli expression system (Tang et al., 1994; Chen et al., 2000). We choose Chn as a model protein for verification effects of Trx on enzymes production because of the presence of disulfide bridges in the enzyme structure and possibility of easy Trx removing from recombinant fusion protein at pH 2.5–3.0 after autocatalytic breaking of the peptide bond in the position F58-G59. That makes this model simple, economical and conventional for checking the enzymatic activity of Chn after refolding.

The aim of this work was to study dynamics of changes in the milk-clotting activity (MA) of rChn – recombinant chymosin (s) synthesized as part of the fusion sequence of recombinant thioredoxin-prochymosin (rTrx-ProChn) or in the form of recombinant prochymosin (rProChn).

## Materials and methods

Construction of plasmids, production, and cultivation of producer strains. Earlier, we constructed a plasmid vector containing the sequence of the alpaca prochymosin gene with optimized codon composition for the E. coli expression system (Belenkaya et al., 2018). To construct a vector containing the thioredoxin sequence, the alpaca prochymosin gene was amplified using a pair of primers Trx-Vic-F 5′-CAGC GGTATTACCAGAATCCCAC-3′ and Trx-Vic-R 5′-AAA AAAAAGCTTCTAAATGGCTTTGGCCAG-3′. Amplification was carried out according to the following program: 95 °C – 5 min (one cycle), 95 °C – 30 s, 59 °C – 30 s, 72 °C – 60 s (30 cycles). The resulting PCR product (1095 bp long) was cloned into the pET32a vector at the unique Msp20I and HindIII restriction sites so that the alpaca prochymosin gene was located in the same reading frame as the thioredoxin gene.

**Fig. 1.**
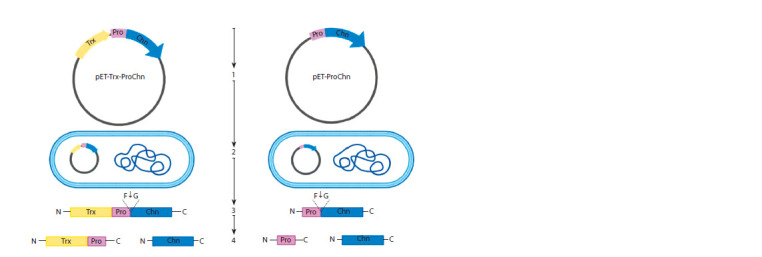
Production and cleavage of rTrx-ProChn and rProChn proteins 1 – pET-Trx-ProChn and pET-ProChn recombinant plasmid construction; 2 – E. coli transformation; 3 – renaturation and activation of rTrx-ProChn and rProChn; 4 – rTrx-ProChn and rProChn cleavage at F58-G59 site.

The E. coli BL21 (DE3) strain (Invitrogen Corp., USA) was chemically transformed with pET-TrxProChn and pETProChn plasmids (Fig. 1)

The culture conditions for both producers and the procedures used for the renaturation of both recombinant proteins were identical. Individual colonies containing pET-TrxProChn and pET-ProChn plasmids were cultured in LB medium containing 100 μg/ml ampicillin overnight on an orbital shaker at 180 rpm and 37 °C. The inoculum (1.0 % v/v) was transferred to an Erlenmeyer flask containing LB medium and grown to an optical density of 0.8 at 600 nm. Next, the inductor, isopropyl- β-D-1-thiogalactopyranoside, was added to the inoculum, to a final concentration of 0.1 mM, and additionally cultured on a shaker (180 rpm) for 6 h at 37 °C.

Isolation and solubilization of inclusion bodies. The cell biomass was separated from the culture medium by centrifugation at 5000 g and 4 °C for 20 min. The bacterial pellet was resuspended in STET buffer (8 % sucrose; 50 mM Tris; 20 mM EDTA; 1 % Triton X-100 pH 8.0) at the rate of 20 ml of buffer per gram of biomass and incubated overnight at 4 °C; at the end of the incubation, the cells were disrupted using an ultrasonic homogenizer Soniprep 150 Plus, and inclusion bodies were pelleted by centrifugation at 20,000 g and 4 °C for 20 min.

The pelleted inclusion bodies were dissolved in Buffer A (50 mM KH2PO4, 150 mM NaCl, pH 10.7) containing 8 M urea, which was added in a ratio of 15 ml of buffer per gram of pellet and incubated for 2 h at 30 °C. The resulting solution containing chymosin was centrifuged at 20,000 g and 4 °C for 15 min. The supernatant was separated and protein concentration was determined by the Bradford method (Bradford, 1976).

Renaturation of the target proteins was performed according to three protocols (Table 1). The supernatant (target protein solution) was diluted with buffer A to a final protein concentration of 180 μg/ml. In protocol No. 1, the resulting solution was incubated for 24 h at 4 °C, in protocols No. 2 and 3, incubation was carried out for 24 h at 15 °C. At the end of incubation, the pH of the solution was adjusted to 8.0 using 1M HCl solution, it was kept for 1 h at 15 °C and dialyzed against buffer B (50 mM Tris HCl, 150 mM NaCl, pH 8.0).

**Table 1.**
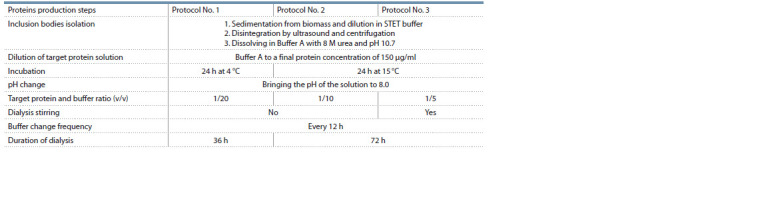
Chymosin proteins production and protocols for renaturation

The ratio of target protein solution volumes and buffer, the duration of dialysis, the frequency of changing the buffer and some other parameters varied depending on the renaturation protocols (see Table 1). As a result, the obtained solutions contained rProChn and rTrx-ProChn variants of the target protein.

Determining the localization of recombinant proteins. The localization of rProChn and rTrx-ProChn proteins was determined by electrophoresis in the presence of sodium dodecyl sulfate (SDS-PAGE) according to the Laemmli method. Mixes of standard proteins from the PageRuler Unstained Protein Ladder kit (Thermo Scientific, USA) were used as molecular weight markers (MW). To determine relative content of the target protein on electrophoregrams, the Gel-Pro Analyzer 3.1 software was used.

Activation of rProChn and rTrx-ProChn. The activation of rProChn and the chimeric protein rTrx-ProChn was carried out in parallel, by the method of stepwise change in pH (Marciniszyn et al., 1976). As a result, samples of rChn alpaca, activated from rProChn (without Trx) – designated as rChn (Trx–), and samples of rChn alpaca, activated from rTrx-ProChn – designated as rChn (Trx+), were obtained.

Evaluation of total and relative milk-clotting activity. A 10.0 % solution of standardized skimmed milk powder (MZSF OJSC, Russia) in 5 mM CaCl2, pH 6.5, was used as a substrate. A 0.5 % aqueous solution of a dry bovine rChn with a certified MA value was used as a control. Prior to determining the MA, the control sample and the liquid preparation of rChn were kept in a water bath at 35 °C for 15 min and cooled to room temperature. The procedure for determining MA was carried out in a water bath at 35 °C. Substrate solution (2.5 ml) was placed into a glass tube and heated at 35 °C for 5 min. An aliquot (0.25 ml) of an enzyme was added to the substrate, a stopwatch was turned on, and the resulting reaction mixture was immediately thoroughly mixed. The time when the first flakes of the coagulated substrate were observed in the drops of the reaction mixture applied onto the tube wall was considered to be the clotting time. The milk-clotting activity was expressed in arbitrary units (AU) per 1 ml (AU/ml) and calculated using the equation: MA (AU/ml) = 0.005*C*T1/T2, C = certified MA value of the control rChn sample in AU per 1 gram, 0.005 = the dilution factor, T1 = coagulation time for the control rChn sample of chymosin, T2 = coagulation time for the test rChn sample. Determination of total MA in each sample was performed in triplicate (n = 3).

Dynamics of relative milk-clotting activity. After activation and determination of the starting MA, samples were stored in plastic tubes at a temperature of 8 ± 1 °C. After 17, 25, 43, 60 days, the relative MA of the samples was determined. To visualize the obtained data, graphs of the dependence of the relative MA on the duration of storage were plotted. The starting MA values were taken as 100 %.

Statistical processing of the obtained data was carried out in the computing environment of an Excel 2007 spreadsheet processor (Microsoft Corporation, USA). For quantitative variables, the results are presented as the arithmetic mean (M) with an indication of standard deviation (± SD). M was used for plotting, values ± SD are given in the tables.

## Results

Construction of plasmids and producer strains

The pET production system was chosen as one of the most convenient systems for expression of recombinant proteins in E. coli (Hayat et al., 2018). A specific feature of this expression system is that the target genes are cloned into specialized pET plasmids under a strong promoter control of bacteriophage T7. The promoter is specifically recognized by T7 RNA polymerase and is not recognized by E. coli RNA polymerases. Thus, expression of the target gene is induced in the presence of a source of T7 RNA polymerase in the host cell. RNA synthesis by T7 RNA polymerase occurs so selectively and efficiently that almost all of the cell’s resources are switched to this process. The content of the target product can reach 50 % of total cellular protein (Chen, 2012).

**Fig. 2.**
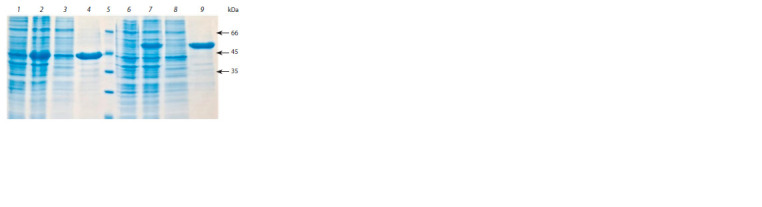
SDS-PAGE analysis of protein preparations from E. coli transfected with pET-ProChn (lanes 1–4) or pET-Trx-ProChn (lanes 6–9). Lanes 1 and 6 – cell biomass before adding the inducer; lanes 2 and 7 – cell biomass 6 h after adding the inducer; lanes 3 and 8 – soluble fraction of cell biomass after treatment with STET buffer and centrifugation; lanes 4 and 9 – insoluble fraction (inclusion bodies) of cell biomass after treatment with buffer A and centrifugation; lane 5 – molecular weight markers.

In our work, the relative content of target proteins (Chn) in case of rProChn producer and rTrx-ProChn was respectively 36 and 19 % of the total amount of protein (Fig. 2, lanes 2 and 7).

Recombinant proteins production

The bacterium E. coli strain BL21 was chosen as the producer of the chimeric protein. The characteristic features of this expression system are: 1) strict control of protein synthesis; 2) high growth rate of culture and high yield of recombinant proteins; 3) high density of viable bacteria in culture; 4) easy of exogenous DNA transformation (Chen, 2012).

To obtain the target protein, E. coli BL21 strain was transformed with the recombinant plasmid pET-TrxProChn. Then induction was performed by adding isopropyl β-D-1- thiogalactopyranoside (IPTG) (Studier et al., 1990). During cultivation, three different temperatures were used, 18, 25 and 37 °C. However, since no differences in the level and localization of the protein between them were observed, all the main experiments on protein production were carried out at 37 °C.

Localization of recombinant proteins

SDS-PAGE electrophoresis of proteins indicates that the target protein is localized in the insoluble fraction of cell biomass in the form of inclusion bodies (see Fig. 2, lanes 4 and 9). Thus, the introduction of the Trx sequence into the structure of the alpaca Chn gene does not lead to the accumulation of the target protein in the periplasmic space.

We have previously shown that when receiving рChn of alpaca, cow and Altai maral in the expression system of E. coli (strain BL21) using the pET21 vector, the target proteins also accumulate in the producer cells in the form of inclusion bodies (Belenkaya et al., 2018, 2020a, b).

Chymosin concentration

The relative content of target proteins in the inclusion body fractions was 82 % for rProChn and 84 % for rTrx-ProChn (see Fig. 2, lanes 4 and 9). After dilution at the refolding stage, the concentration of rProChn and rTrx-ProChn in the solution was ≈150 μg/ml. The actual MW of rProChn and rTrx-ProChn differed by 1.3 times and amounted to 40.5 kDa and 52.5 kDa, respectively, which corresponded to predicted values. Therefore, to calculate the relative MA of Сhn derived from rTrx-ProChn rChn (Trx+), we used a lowering factor of 1.3 to reflect the actual enzyme concentration. Consequently, for the calculation of the relative MA of rChn (Trx–) we used concentration of 150 μg/ml while for rChn (Trx+) we considered actual enzyme concentration at 114 μg/ml.

Activation of recombinant proteins

To prevent the autocatalytic activation of the target proteins, after the completion of refolding, the rProChn and rTrx- ProChn samples were stored at pH 8.0 (buffer B). To activate zymogens, the titration procedure to pH = 3.0 was chosen, since in preliminary experiments maximum MA values were observed at this pH. The final pH value of 5.8 was selected as chymosin is stable at moderately acidic pH values = 5.3–6.3 and loses activity at pH > 6.5 (http://www.brenda-enzymes. org/enzyme.php?ecno=3.4.23.4). Before the start of activation, MA of the samples was < 1.0 CU/ml.

Data presented in Table 2 indicate that immediately after activation, the relative МА of the rChn (Trx–) and rChn (Trx+) samples increases by more than 700 times, which indicates the efficiency of the procedure of zymogen activation.

**Table 2.**
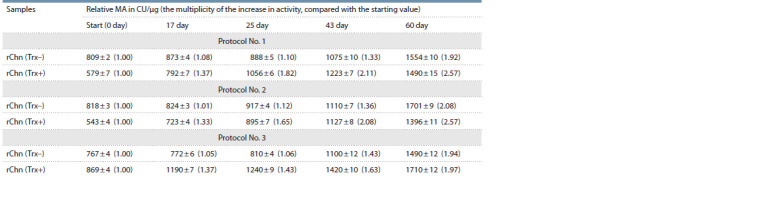
Changes in the relative MA of rChn (Trx–) and rChn (Trx+) obtained using the renaturation protocols No. 1–3 Note. MA – milk-clotting activity; CU – conventional units.

Influence of refolding parameters on the activity of Chns, derived from rProChn and rTrx-ProChn

The MA changes in the rChn (Trx–) and rChn (Trx+) samples was monitored in order to track the possible prolonged effect of Trx on the activity of marker enzymes after refolding and activation of zymogens

Using refolding protocol No. 1 and 2, starting (immediately after activation) and finishing (after long-term storage) values of the MA in rChn (Trx–) were higher than in rChn (Trx+). At the same time, final coagulation abilities of rChn (Trx–) and rChn (Trx+) were different (see Тable 2). After 60 days of incubation, the relative MA of rChn (Trx–) increased by 1.9–2.1 times, while the activity of the rChn (Trx+) samples, during the same time, increased by more than 2.5 times.

The main difference of protocol No. 3 from protocols No. 1 and No. 2 was the reduced ratio of the target protein and dialysis buffer volumes (see Table 1). It is curious that under the conditions of protocol No. 3, the efficiency of refolding of the rProChn sample decreases and turns out to be lower than when using refolding protocols No. 1 and 2 (see Тable 2). Immediately after activation, the relative MA of the rChn (Trx+) sample, renatured according to protocol No. 3, was significantly ( р > 0.05) higher than activity of rChn (Trx–) obtained using protocols No. 2 and 1.

Utilization of the renaturation protocol No. 3 makes it possible to increase efficiency of the rTrx-ProChn refolding stage (see Table 2). As a consequence, immediately after activation of zymogens and after 60 days of incubation, the values of the relative MA in case of the rChn (Trx+) were 13 and 15 % higher than in the rChn (Trx–). This allows us to conclude that in the expression system used by us, application of Trx under certain refolding conditions (protocol No. 3) makes it possible to elevate the MA of rChn (Trx+) both immediately after the activation of the zymogen and in the process of long-term storage of the enzyme

**Fig. 3.**
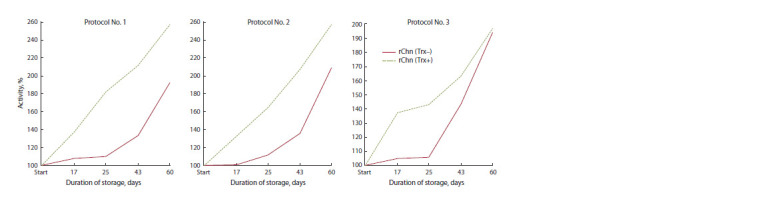
Dependency of the relative MA on the duration of storage of rChn (Trx–) and rChn (Trx+), obtained using the renaturation protocols No. 1–3.

During long-term storage, there was a constant increase in the MA of rChn (Trx–) and rChn (Trx+) samples, probably due to refolding of a part of the enzyme molecules that did not keep the correct tertiary structure after the renaturation procedure. To analyze the dynamics of MA in the rChn (Trx–) and rChn (Trx+) obtained using different renaturation protocols, a graph of MA (%) versus storage duration was plotted. The data presented in Fig. 3 show that the dynamics of MA changes are different, especially in the first 25–43 days of storage. Since MA of rChn (Trx+) is ahead of rChn (Trx+) in terms of growth dynamics, it can be assumed that the introduction of Trx into the structure of zymogen additionally stimulates the refolding of the enzyme during long-term storage.

## Conclusion

Thus, it was found that in the E. coli expression system (strain BL21), attachment of Trx to the N-terminal region of ProChn in alpaca provides accumulation of the target protein exclusively in the form of inclusion bodies.

Coexpression of thioredoxin and prochymosin in the composition of the chimeric molecule rTrx-ProChn and optimization of the conditions of zymogen refolding make it possible to increase the yield of alpaca rСhn immediately after activation and during long-term storage by ~13 and ~15 %, respectively.

## Conflict of interest

The authors declare no conflict of interest.
